# Naringenin, a Food Bioactive Compound, Reduces Oncostatin M Through Blockade of PI3K/Akt/NF-κB Signal Pathway in Neutrophil-like Differentiated HL-60 Cells

**DOI:** 10.3390/foods14010102

**Published:** 2025-01-02

**Authors:** Na-Ra Han, Hi-Joon Park, Seong-Gyu Ko, Phil-Dong Moon

**Affiliations:** 1 College of Korean Medicine, Kyung Hee University, Seoul 02447, Republic of Korea; nrhan@khu.ac.kr; 2 Korean Medicine-Based Drug Repositioning Cancer Research Center, College of Korean Medicine, Kyung Hee University, Seoul 02447, Republic of Korea; epiko@khu.ac.kr; 3 Department of Anatomy & Information Sciences, College of Korean Medicine, Kyung Hee University, Seoul 02447, Republic of Korea; acufind@khu.ac.kr; 4 Department of Preventive Medicine, College of Korean Medicine, Kyung Hee University, Seoul 02447, Republic of Korea; 5 Center for Converging Humanities, Kyung Hee University, Seoul 02447, Republic of Korea

**Keywords:** Oncostatin M, Naringenin, Phosphatidylinositol 3-kinase, Akt, nuclear factor-κB

## Abstract

Oncostatin M (OSM) plays a crucial role in diverse inflammatory reactions. Although the food bioactive compound naringenin (NAR) exerts various useful effects, including antitussive, anti-inflammatory, hepatoprotective, renoprotective, antiarthritic, antitumor, antioxidant, neuroprotective, antidepressant, antinociceptive, antiatherosclerotic, and antidiabetic effects, the modulatory mechanism of NAR on OSM expression in neutrophils has not been specifically reported. In the current work, we studied whether NAR modulates OSM release in neutrophil-like differentiated (d)HL-60 cells. To assess the modulatory effect of NAR, enzyme-linked immunosorbent assay (ELISA), quantitative real-time polymerase chain reaction (qRT-PCR), Western blotting, and immunofluorescence assay were employed. While exposure to granulocyte-macrophage colony-stimulating factor (GM-CSF) induced elevated OSM release and mRNA expression, the elevated OSM release and mRNA expression were diminished by the addition of NAR in dHL-60 cells. While the phosphorylation of phosphatidylinositol 3-kinase, protein kinase B (Akt), and nuclear factor (NF)-κB was upregulated by exposure to GM-CSF, the upregulated phosphorylation was inhibited by the addition of NAR in dHL-60 cells. Consequently, the results indicate that the food bioactive compound NAR may have a positive effect on health (in health promotion and improvement) or may play a role in the prevention of inflammatory diseases.

## 1. Introduction

Oncostatin M (OSM) was first identified as a growth regulator of tumor cells [1]. Numerous studies were thus focused on cancer research and reported that the levels of OSM were upregulated in various patients with cancer as well as tumor tissues [2,3,4]. On the other hand, recent studies reported that OSM is a proinflammatory factor and is released in a variety of cells, inclusive of dendritic cells, stimulated T cells, macrophages, monocytes, and neutrophils [5,6,7,8,9].

Many research groups have reported that OSM is implicated with various pathophysiologic processes, tumor progression, extracellular matrix reorganization, the formation of blood, hepatic regeneration, heart remodeling, and inflammation [2,10,11,12,13]. Among them, inflammation is an important area where OSM plays various roles [2]. OSM, a proinflammatory cytokine, is essential in inflammatory responses in rheumatoid arthritis and liver diseases [2,14]. In addition, several studies reported that OSM plays a role in pulmonary inflammatory disorders [15,16]. West and colleagues reported that the addition of human OSM leads to elevated inflammatory responses in the human normal intestinal stroma [5]. Exposure to OSM protein led to augmented inflammatory responses in HaCaT keratinocytes [9]. Our previous results showed that exposure to recombinant OSM resulted in augmented IL-1β production in human HaCaT keratinocytes, indicating that OSM plays a role in inflammatory reactions [17]. It is also known that OSM is mainly released in neutrophil cells in diseases of the airway [16]. In general, HL-60 has been used as a representative cell line to investigate neutrophils and their properties due to the short life span and donor variability of primary neutrophil cells [17]. Differentiation into neutrophil-like cells resulted from exposure to dimethyl sulfoxide (DMSO) in HL-60 cells [18,19]. The functions of neutrophil cells were investigated in many studies using neutrophil-like differentiated (d)HL-60 cells by means of DMSO [18,20]. Furthermore, the regulatory mechanism of OSM by naringenin (NAR) in neutrophils is still unclear. We thus examined the mechanism involved in OSM inhibition by NAR in neutrophil-like dHL-60 cells.

It has been reported that phosphatidylinositol 3-kinase (PI3K) plays a crucial role in the regulation of diverse signal processes inside cells [21]. A downstream factor of PI3K is Akt kinase, which is a crucial factor in inflammatory reactions [22]. The PI3K/Akt signal pathway accounted for a significant portion of the regulation of cytokine production [23]. In various pathological conditions, such as tumorigenesis, heart diseases, and inflammatory diseases, the PI3K/AKT signal pathway plays an essential role [24]. Activation of nuclear factor (NF)-κB, which is a downstream agent of Akt, resulted from Akt activation [23]. NF-κB is a well-known agent in controlling inflammatory processes [25]. Su and colleagues reported that PI3K/Akt/NF-κB signaling controls the expression of mRNA and the production of OSM [25].

Naringenin (NAR) is a food bioactive compound that is contained in tomatoes and citrus fruits [26]. NAR exerts a wide variety of effects including antitussive, anti-inflammatory, hepatoprotective, renoprotective, antiarthritic, antitumor, antioxidant, neuroprotective, antidepressant, antinociceptive, antiatherosclerotic, and antidiabetic effects [27,28,29,30,31,32,33,34]. However, the underlying action mechanism of NAR in OSM release in neutrophil cells should be clarified. Here, we studied whether NAR modulates OSM production in dHL-60 cells. In our previous report, we examined when each factor reaches a peak in dHL-60 cells. The phosphorylated (p)-PI3K reached the maximum level 15 min after GM-CSF stimulation; p-Akt, 30 min; p-p65, 30 min; OSM mRNA, 30 min; and OSM protein, 4 h [17]. Therefore, we investigated whether NAR could affect PI3K/Akt/NFκB/OSM mRNA/OSM protein signaling cascades.

## 2. Materials and Methods

### 2.1. Reagents

NAR (C_15_H_12_O_5_) was procured from Sigma-Aldrich Inc. (St. Louis, MO, USA). Recombinant human GM-CSF and OSM capture and detection antibodies were obtained from R&D Systems (Minneapolis, MN, USA). Phosphorylated (p)-PI3K p85 was obtained from Cell Signaling Technology (Danvers, MA, USA). All Western blotting antibodies, excepting p-PI3K p85, were prepared by Santa Cruz Biotechnology (Santa Cruz, CA, USA).

### 2.2. Cultivation and Treatments of Cells

HL-60 cells were procured from Korean Cell Line Bank (Seoul, Republic of Korea) and maintained in RPMI 1640 (Gibco, Grand Island, NY, USA) with 10% FBS. To differentiate them into neutrophil-like cells, HL-60 cells were maintained in a medium containing DMSO (1.3%) for seven days. In addition, dHL-60 cells were exposed to GM-CSF (5 ng/mL), as described previously [17,35].

### 2.3. Cell Viability

dHL-60 cells (5 × 10^4^ cells in 500 μL of medium) were seeded in a 24-well plate and incubated with NAR for 1 h. Subsequently, 5 ng/mL of GM-CSF was added, and the cells were incubated for 4 h. The cytotoxicity was assessed, as described previously [36,37].

### 2.4. ELISA of OSM

dHL-60 cells (2.5 × 10^5^ cells in 500 μL of medium) were seeded in a 24-well plate and incubated with NAR for 1 h. Subsequently, 5 ng/mL of GM-CSF was added, and the cells were incubated for 4 h. OSM release was analyzed by means of ELISA, as previously described [38,39,40,41].

### 2.5. qRT-PCR

dHL-60 cells (2 × 10^6^ cells in 2 mL of medium) were seeded in a 6-well plate and incubated with NAR for 1 h. Subsequently, 5 ng/mL of GM-CSF was added, and the cells were incubated for 30 min. The expression of target genes was detected by Applied Biosystems (Foster City, CA, USA) and Power SYBR^®^ Green Master Mix (Applied Biosystems, Foster City, CA, USA), as described previously [42,43]. PCR was performed with the following primers for human OSM (5′-GCTCACACAGAGGACGCTG-3′, 5′-GGAGCACGCGGTACTCTTTC-3′) and GAPDH (5′-TCGACAGTCAGCCGCATCTTCTTT-3′, 5′-ACCAAATCCGTTGACTCCGACCTT-3′).

### 2.6. Western Blotting

dHL-60 cells (1 × 10^7^ cells in 2 mL of medium) were seeded in a 6-well plate and incubated with NAR for 1 h. Subsequently, 5 ng/mL of GM-CSF was added, and the cells were incubated for each condition (PI3K-15 min; Akt-30 min; NF-κB-30 min). Western blotting was performed, as described previously [44,45,46].

### 2.7. Fluorescence Microscopy

dHL-60 cells (2 × 10^6^ cells in 2 mL of medium) were seeded in a 6-well plate and incubated with NAR for 1 h. Subsequently, 5 ng/mL of GM-CSF was added, and the cells were incubated for 30 min. Fluorescence microscopy was performed, as previously described [47,48].

### 2.8. Statistical Methods

Statistical analyses were carried out with the SPSS software program (version 25, Armonk, NY, USA). Measurement data were analyzed with the independent samples *t*-test and one-way analysis of variance (ANOVA). * *p* < 0.05 signifies significant differences.

## 3. Results

### 3.1. Downregulation of OSM Production by NAR in dHL-60 Cells

**Figure 1:**
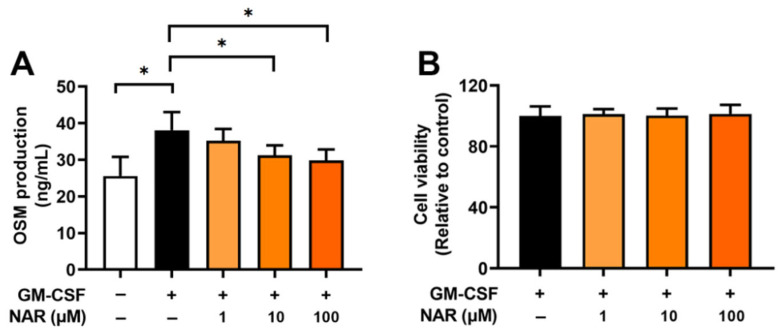
Inhibitory effects on OSM production by NAR and cytotoxic effects on dHL-60 cells. (**A**) The cells (2.5 × 10^5^ cells in 500 μL of medium) were exposed to recombinant human GM-CSF (5 ng/mL) for 4 h after pretreatment with NAR (1, 10, and 100 μM) for 1 h. OSM production was measured by ELISA. (**B**) The cells (5 × 10^4^ cells in 500 μL of medium) were cultured with NAR (1, 10, and 100 μM) for 4 h. The cytotoxicity of NAR on dHL-60 cells was determined by an MTT assay. Data are the mean ± SD of three independent experiments. * *p* < 0.05 signifies significant differences.

To determine the effect of NAR on OSM production, dHL-60 cells were stimulated with GM-CSF for 4 h after preincubation with NAR (1, 10, and 100 μM) or PBS for 1 h. In our previous report, the OSM levels reached the maximum production 4 h after GM-CSF addition [17]. Similarly to our previous report [17], the addition of GM-CSF led to elevated OSM levels. The elevated OSM levels were dose-dependently attenuated by the addition of NAR to dHL-60 cells (Figure 1A). When the cells were preincubated with various concentrations of NAR (1, 10, and 100 μM), decreased OSM levels were shown (i.e., 35.267 ± 3.167, 31.283 ± 2.696, and 29.867 ± 2.998, respectively). The GM-CSF-stimulated group after preincubation with PBS showed the highest OSM value (38.117 ± 4.944), while the unstimulated group after preincubation with PBS showed the lowest OSM value (25.633 ± 5.161). The MTT assay showed that NAR was not toxic to dHL-60 cells, as cell viability remained unaffected at various concentrations of NAR (1, 10, and 100 μM, Figure 1B). To obtain more profound insights into the effect of NAR on OSM protein levels, we pretreated NAR for 1, 12, and 24 h. As shown in Figure S1, there was no significant difference between these times; however, 24 h pretreatment showed greater inhibition than 1 h pretreatment, but not at a significant level. To support cell viability data, we examined the cytotoxicity of NAR when treated alone. There was no cytotoxicity with NAR-alone treatment (Figure S2).

### 3.2. Downregulation of OSM mRNA Expression by NAR in dHL-60 Cells

**Figure 2:**
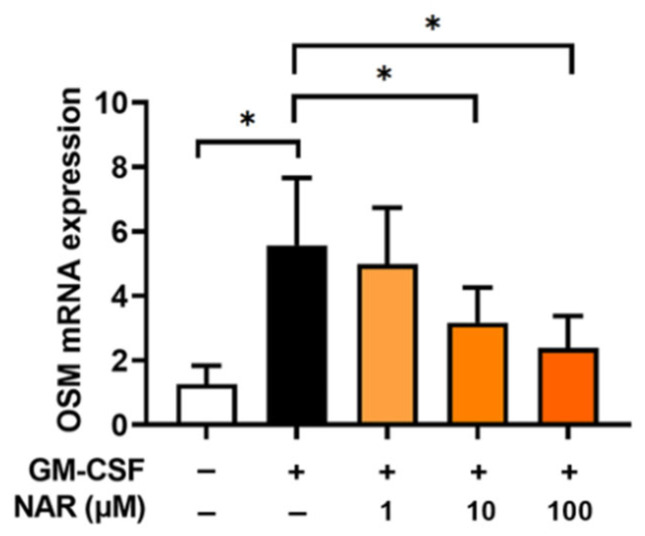
Inhibitory effects on OSM mRNA expression by NAR in dHL-60 cells. The cells (2 × 10^6^ cells in 2 mL of medium) were exposed to recombinant human GM-CSF (5 ng/mL) for 30 min after pretreatment with NAR (1, 10, and 100 μM) for 1 h. Total RNA was isolated and OSM expression was analyzed by means of qRT-PCR. Data are the mean ± SD of three independent experiments. * *p* < 0.05 signifies significant differences.

To assess the effect of NAR on OSM mRNA expression, dHL-60 cells were stimulated with GM-CSF for 30 min after preincubation with NAR (1, 10, and 100 μM) or PBS for 1 h. In our previous report, the OSM mRNA levels reached the maximum expression 30 min after GM-CSF addition [17]. Similarly to our previous work [17], the addition of GM-CSF resulted in increased OSM mRNA expression. The increased OSM mRNA expression was dose-dependently attenuated by the addition of NAR in dHL-60 cells (Figure 2). When the cells were preincubated with various concentrations of NAR (1, 10, and 100 μM), decreased OSM mRNA expression was shown (i.e., 4.992 ± 1.745, 3.172 ± 1.089, and 2.387 ± 0.991, respectively). The GM-CSF-stimulated group after preincubation with PBS showed the highest OSM mRNA expression (5.570 ± 2.096), while the unstimulated group after preincubation with PBS showed the lowest OSM mRNA expression (1.262 ± 0.579). Subsequent assays (i.e., Western blotting and immunofluorescence assay) were conducted at a concentration of 100 μM of NAR, as 100 μM of NAR showed the most inhibitory activity. To obtain more profound insight into the effect of NAR on OSM mRNA expression, we pretreated NAR for 1, 12, and 24 h. As shown in Figure S3, there was no significant difference between these times; however, 24 h pretreatment showed more inhibition than 1 h pretreatment, but not at a significant level.

### 3.3. Inhibition of PI3K Phosphorylation by NAR in dHL-60 Cells

**Figure 3:**
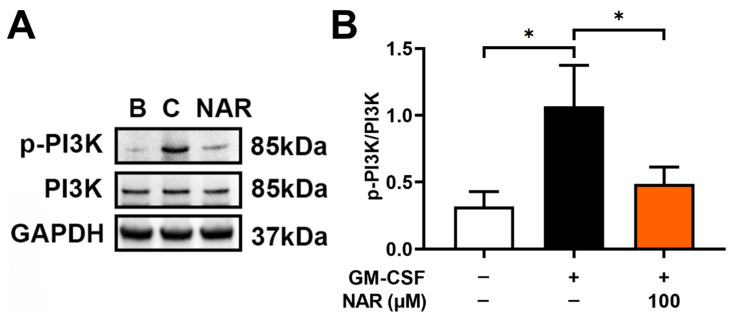
Inhibitory effect on PI3K phosphorylation by NAR in dHL-60 cells. (**A**) The cells (1 × 10^7^ cells in 2 mL of medium) were exposed to recombinant human GM-CSF (5 ng/mL) for 15 min after pretreatment with NAR (100 μM) or PBS for 1 h. B—PBS-treated and unstimulated cells; C—PBS-treated and GM-CSF-stimulated cells. (**B**) Each band was quantitated using the ImageJ software program (https://imagej.net/ij/). Data are the mean ± SD of three independent experiments. * *p* < 0.05 signifies significant differences.

To analyze the modulatory mechanism of OSM decrement by NAR, dHL-60 cells were exposed to GM-CSF for 15 min after preincubation with NAR (100 μM) or PBS for 1 h. In our previous report, PI3K reached the maximum phosphorylation 15 min after GM-CSF addition [17]. Similarly to our previous research [17], the addition of GM-CSF showed increased PI3K phosphoryation; however, the increased PI3K phosphorylation was diminished by the addition of NAR in dHL-60 cells (Figure 3). To obtain further information on the effect of NAR on PI3K phosphorylation, we pretreated NAR for 1, 12, and 24 h. As shown in Figure S4, there was no significant difference between these times.

### 3.4. Inhibition of Akt Phosphorylation by NAR in dHL-60 Cells

**Figure 4:**
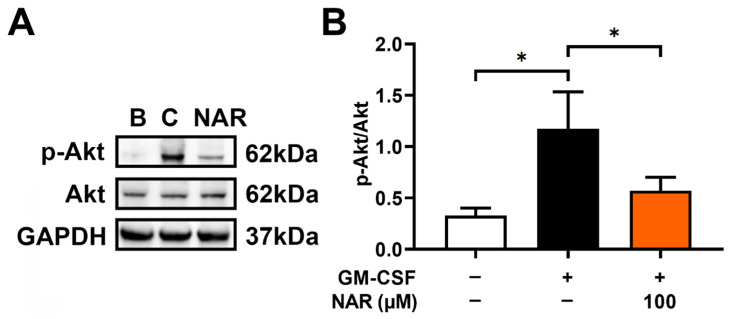
Inhibitory effect on Akt phosphorylation by NAR in dHL-60 cells. (**A**) The cells (1 × 10^7^ cells in 2 mL of medium) were exposed to recombinant human GM-CSF (5 ng/mL) for 30 min after pretreatment with NAR (100 μM) for 1 h. B—PBS-treated and unstimulated cells; C—PBS-treated and GM-CSF-stimulated cells. (**B**) Each band was quantitated using the ImageJ software program. Data are the mean ± SD of three independent experiments. * *p* < 0.05 signifies significant differences.

To identify the modulatory mechanism of OSM downregulation by NAR, dHL-60 cells were stimulated with GM-CSF for 30 min after preincubation with NAR (100 μM) for 1 h. In our previous report, Akt reached the maximum phosphorylation 30 min after GM-CSF addition [17]. Similarly to our previous report [17], the addition of GM-CSF induced upregulated Akt phosphorylation (Figure 4A). The upregulated Akt phosphorylation was downregulated by the addition of NAR in dHL-60 cells (Figure 4).

### 3.5. Inhibition of NF- κB Phosphorylation (p-p65) by NAR in dHL-60 Cells

**Figure 5:**
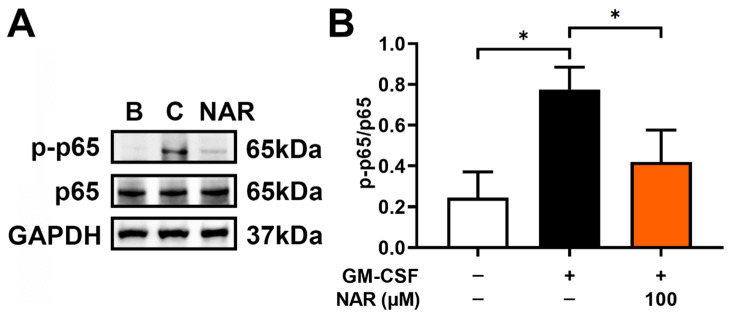
Inhibitory effect on NF-κB phosphorylation (p-p65) by NAR in dHL-60 cells. (**A**) The cells (1 × 10^7^ cells in 2 mL of medium) were exposed to recombinant human GM-CSF (5 ng/mL) for 30 min after pretreatment with NAR (100 μM) for 1 h. B—PBS-treated and unstimulated cells; C—PBS-treated and GM-CSF-stimulated cells. (**B**) Each band was quantitated using the ImageJ software program. Data are the mean ± SD of three independent experiments. * *p* < 0.05 signifies significant differences.

To understand the modulatory mechanism of OSM downregulation by NAR, dHL-60 cells were stimulated with GM-CSF for 30 min after preincubation with NAR (100 μM) for 1 h. In our previous report, NF-κB reached the maximum phosphorylation 30 min after GM-CSF addition [17]. Similarly to our previous research [17], elevated NF-κB phosphorylation (p-p65) resulted from the addition of GM-CSF (Figure 5A). The elevated NF-κB phosphorylation (p-p65) was reduced by the addition of NAR in dHL-60 cells (Figure 5).

### 3.6. Inhibition of p-NF-κB Fluorescence (p-p65) Expression by NAR in dHL-60 Cells

**Figure 6:**
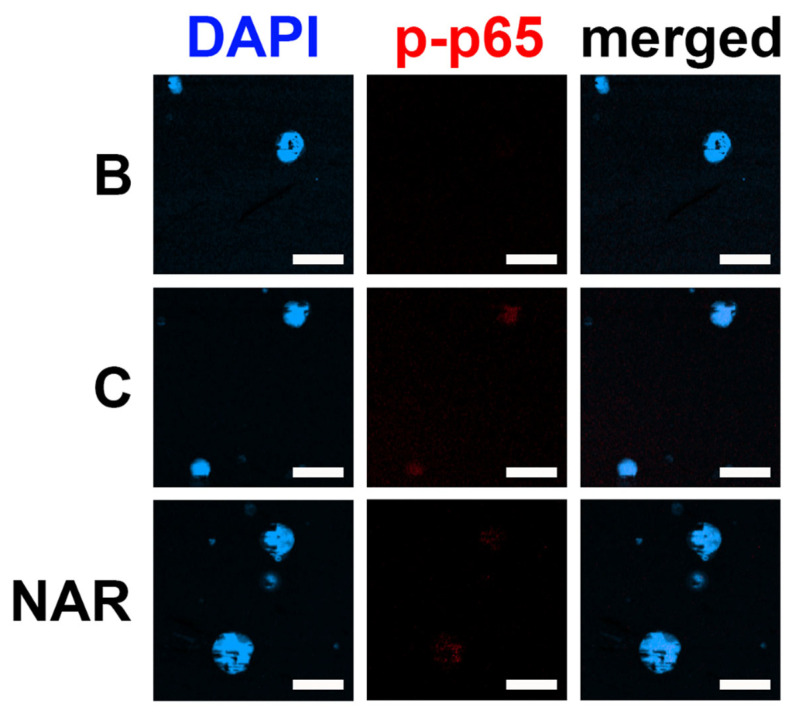
Inhibitory effect on p-NF-κB (p-p65) immunofluorescence by NAR in dHL-60 cells. The cells (2 × 10^6^ cells in 2 mL of medium) were exposed to recombinant human GM-CSF (5 ng/mL) for 30 min after pretreatment with NAR (100 μM) for 1 h. B—PBS-treated and unstimulated cells; C—PBS-treated and GM-CSF-stimulated cells. Fluorescence microscopy image of representative cells in each treatment group is shown (scale bar = 20 μm).

To confirm the modulatory processes of NAR in fluorescence expression, an immunofluorescence assay for p-NF-κB (an essential and last step in PI3K/Akt/NF-κB signal cascades) was performed in dHL-60 cells. The cells were activated with GM-CSF for 30 min after preincubation with NAR (100 μM) for 1 h. As demonstrated in a previous study [17], upregulated NF-κB phosphorylation (p-p65) resulted from the addition of GM-CSF (Figure 6). The upregulated NF-κB phosphorylation (p-p65) was diminished by the addition of NAR in dHL-60 cells (Figure 6). To confirm that NAR-mediated effects were due to the PI3K/AKT/NF-κb signaling axis, PI3K inhibitor wortmannin (the first factor of the PI3K/AKT/NF-κb signaling axis) was used. As shown in Figure S5, OSM production was suppressed by PI3K inhibitor wortmannin treatment.

## 4. Discussion

It has been reported that inflammatory diseases, such as chronic rhinosinusitis and asthma, show high expression of OSM [16,49,50]. Ma and colleagues [51] suggested that the addition of GM-CSF results in upregulated mRNA expression of OSM. Furthermore, several studies reported that exposure to GM-CSF induces upregulated OSM expression in human neutrophil cells [16,35,52,53]. As also demonstrated in a previous study [17], exposure to GM-CSF led to augmented mRNA and protein levels of OSM in the present study (Figure 1 and Figure 2). In our previous study [17], we examined the effect of dexamethasone, which is a prescription drug, on OSM expression. In OSM production levels, the inhibitory effect of NAR was somewhat similar to that of dexamethasone. A similar effect to that of dexamethasone thus potentially could be obtained from NAR-containing foods, such as tomatoes and citrus fruits, without a doctor’s prescription. The elevated mRNA expression and protein levels of OSM were attenuated by preincubation with NAR in the present study (Figure 1 and Figure 2). It was reported that treatment with OSM protein leads to elevated inflammatory cell accumulation and increases in inflammatory cytokines and chemokines in murine models [54]. Modur and colleagues [55] suggested that subcutaneous treatment with OSM results in intensified skin inflammatory responses in mice. OSM-overexpressed lung tissues expressed upregulated inflammatory responses in a murine model [56]. Patients with asthma also exhibited high expressions of OSM, whereas there was no expression of OSM in control subjects [50]. Furthermore, blockades of OSM, such as OSM neutralization and an OSM deleted mouse model, exhibited decreased inflammatory levels in mouse colon samples [5]. We thus assume that NAR may be useful to prevent inflammatory diseases or may have a positive effect on health (in health promotion and improvement) through a blockade of OSM.

Generally, PI3K/Akt signaling cascades are crucial in modulating inflammatory responses [21,22,23,24]. Su and colleagues suggested that NF-κB is an important factor in inflammatory reactions [25]. Additionally, OSM production was modulated by PI3K/Akt/NF-κB signal cascades in MG-63 cells [25]. As demonstrated in a previous report [17], OSM production was also modulated by PI3K/Akt/NF-κB signaling pathways in dHL-60 cells. Treatment with LY294002 (a PI3K inhibitor) resulted in decreased levels of diverse inflammatory molecules, such as IL-1β, IL-6, and TNF-α [57]. In addition, the inhibition of PI3K/Akt signaling processes induced an attenuation of joint disease in mice [58]. Several reports suggested that the application of a wide range of PI3K inhibitors (i.e., wortmannin, LY-294002, and IC87114) led to downregulated respiratory inflammatory reactions in mice [59,60]. Treatment with Akt inhibitor induced repressed respiratory inflammatory reactions in a murine model [61]. Several reports suggested that the inhibition of NF-κB leads to repressed respiratory inflammatory reactions in an asthma mouse model [61,62]. Our findings revealed that treatment with NAR suppresses phosphorylated levels of PI3K, Akt, and NF-κB (Figure 3, Figure 4, Figure 5 and Figure 6). Thus, we could presume that OSM inhibition by NAR may be implicated in PI3K/Akt/NF-κB signal cascades in dHL-60 cells. Koch and colleagues suggested that NAR might bind to PI3K and might thus be able to reduce PI3K activity [63]. Therefore, we could assume that NAR might bind to PI3K and might thus be able to inhibit the phosphorylation of PI3K, because NAR reduced PI3K phosphorylation in dHL-60 cells in the present study (Figure 3). To derive more profound information on the effect of NAR on OSM protein and mRNA expression, we pretreated NAR for 1, 12, and 24 h. While there was no significant difference between these times, 24 h pretreatment showed greater inhibition than 1 h pretreatment, but not at a significant level. Thus, 24 h pretreatment could be considered an advanced approach for sample treatment in future studies.

Finally, toxic side effects were not found in SD rats, which were administered 50 mg/kg of NAR for 21 consecutive days [64]. In the current work, we pretreated 100 μM of NAR (approximately 27.225 mg/kg). Therefore, it is believed that NAR might not be toxic to humans at a dose of 100 μM, because 100 µM of NAR is about half of the aforementioned 50 mg/kg NAR dose [64].

## 5. Conclusions

**Figure 7:**
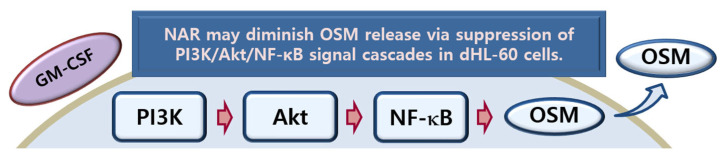
Schematic diagram of OSM inhibition by NAR.

Our findings demonstrate that NAR inhibits OSM release via the suppression of PI3K/Akt/NF-κB signal cascades in dHL-60 cells (Figure 7). The obtained data suggest that the food bioactive compound NAR may have a positive effect on health (in health promotion and improvement) or may play a role in the prevention of inflammatory diseases. Our results are thus expected to provide insights into the health benefits and functional potential of food bioactive compounds. However, there is a limitation in the present study in that an in vivo study was not conducted. Our investigation only focused on the in vitro effect of NAR on the production of OSM in neutrophil-like differentiated cells. Nevertheless, our results may offer base-line data for future in vivo research to clarify the effect of NAR in OSM-related health problems.

## Data Availability

The original contributions presented in this study are included in the article/Supplementary Materials. Further inquiries can be directed to the corresponding author.

## Supplementary Materials

The following supporting information can be downloaded at: https://www.mdpi.com/article/10.3390/foods14010102/s1, Figure S1. Inhibitory effects on OSM production by NAR in dHL-60 cells; Figure S2. Cytotoxic effect by NAR alone on dHL-60 cells; Figure S3. Inhibitory effects on OSM mRNA expression by NAR in dHL-60 cells; Figure S4. Inhibitory effect on PI3K phosphorylation by NAR in dHL-60 cells; Figure S5. Inhibitory effects on OSM production by Wortmannin (PI3K inhibitor) in dHL-60 cells.
