# Supplementary material for: Naringenin, a Food Bioactive Compound, Reduces Oncostatin M Through Blockade of PI3K/Akt/NF-κB Signal Pathway in Neutrophil-like Differentiated HL-60 Cells
Source: Foods. 2025 Jan 2;14(1):102. doi: 10.3390/foods14010102 (PMC11719654; doi:10.3390/foods14010102)
Supplement: Supplementary file 1 [file foods-14-00102-s001.zip › foods-3338587-supplementary.pdf]

foods
MDPI

Supplementary

Naringenin, a Food Bioactive Compound, Reduces Oncostatin M Through Blockade of PI3K/Akt/NF-κB Signal Pathway in Neutrophil-like Differentiated HL-60 Cells

Na-Ra Han [1,2], Hi-Joon Park [3], Seong-Gyu Ko [2,4] and Phil-Dong Moon [5,*]

[1] College of Korean Medicine, Kyung Hee University, Seoul 02447, Republic of Korea; nrhan@khu.ac.kr
[2] Korean Medicine-Based Drug Repositioning Cancer Research Center, College of Korean Medicine, Kyung Hee University, Seoul 02447, Republic of Korea; epiko@khu.ac.kr
[3] Department of Anatomy & Information Sciences, College of Korean Medicine, Kyung Hee University, Seoul 02447, Republic of Korea; acufind@khu.ac.kr
[4] Department of Preventive Medicine, College of Korean Medicine, Kyung Hee University, Seoul 02447, Republic of Korea
[5] Center for Converging Humanities, Kyung Hee University, Seoul 02447, Republic of Korea
* Correspondence: pdmoon@khu.ac.kr; Tel.: +82-2-961-0897

Results

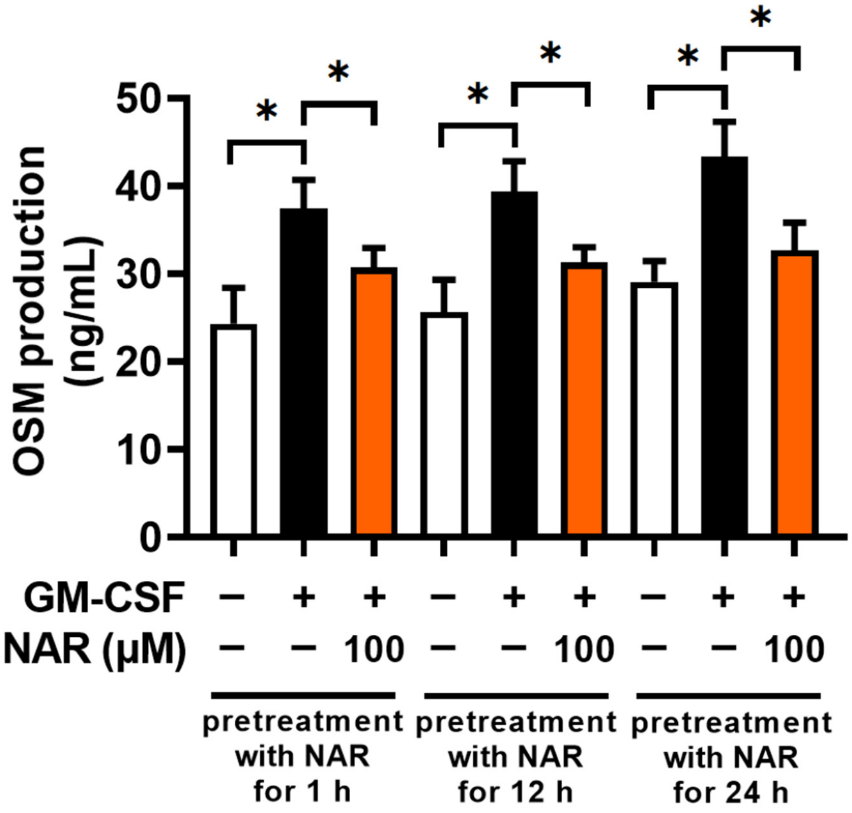

Figure S1. Inhibitory effects on OSM production by NAR in dHL-60 cells. The cells (2.5×10^5 cells in 500 μL of medium) were exposed to recombinant human GM-CSF (5 ng/mL) for 4 h after pretreatment with NAR (100 μM) for 1, 12, and 24 h. OSM production was measured by ELISA. Data are the mean ± SD of three independent experiments. *$p < 0.05$ signifies significant differences.

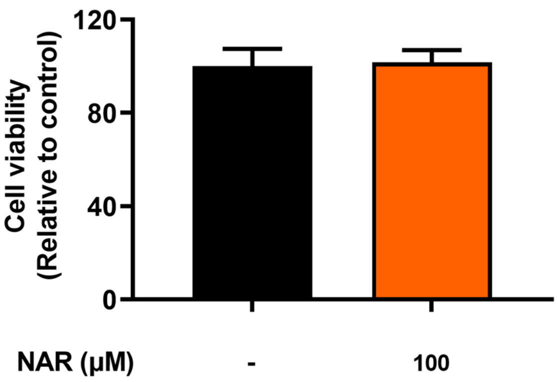

Figure S2. Cytotoxic effect by NAR alone on dHL-60 cells. The cells (5×10^4 cells in 500 μL of medium) were cultured with NAR (100 μM) for 4 h. The cytotoxicity of NAR on dHL-60 cells was determined by an MTT assay. Data are the mean ± SD of three independent experiments.

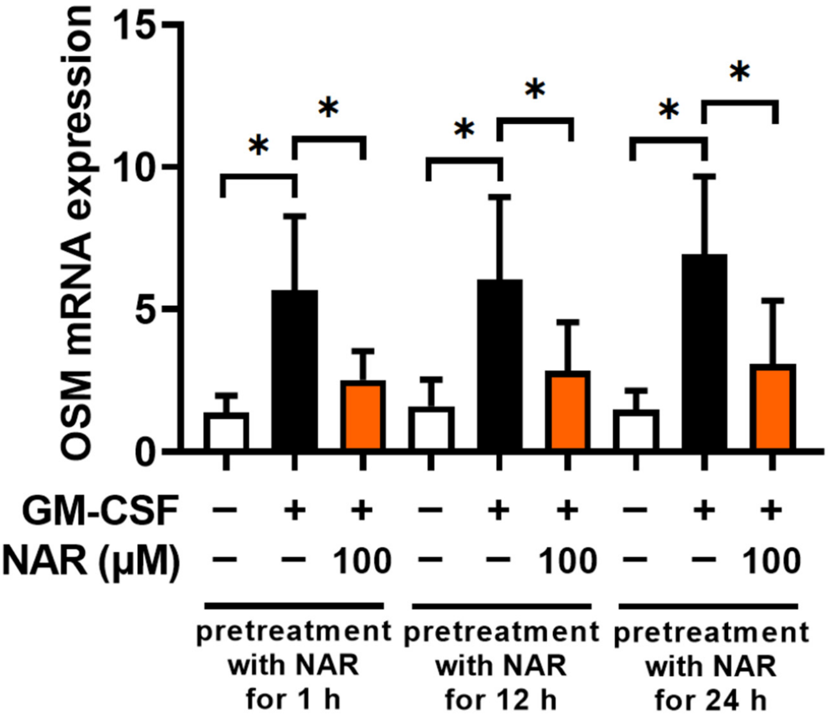

Figure S3. Inhibitory effects on OSM mRNA expression by NAR in dHL-60 cells. The cells (2×10^6 cells in 2 mL of medium) were exposed to recombinant human GM-CSF (5 ng/mL) for 30 min after pretreatment with NAR (100 μM) for 1, 12, and 24 h. Total RNA was isolated and OSM expression was analyzed by means of qRT-PCR. Data are the mean ± SD of three independent experiments. *$p < 0.05$ signifies significant differences.

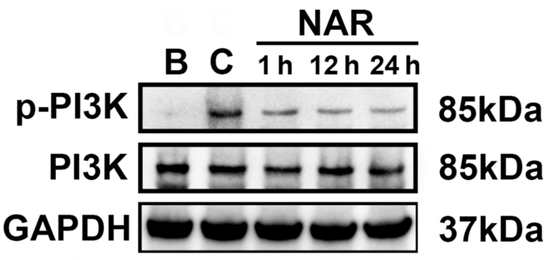

Figure S4. Inhibitory effect on PI3K phosphorylation by NAR in dHL-60 cells. (A) The cells (1×10^7 cells in 2 mL of medium) were exposed to recombinant human GM-CSF (5 ng/mL) for 15 min after pretreatment with NAR (100 μM) for 1, 12, and 24 h. B—PBS-treated and unstimulated cells; C—PBS-treated and GM-CSF-stimulated cells.

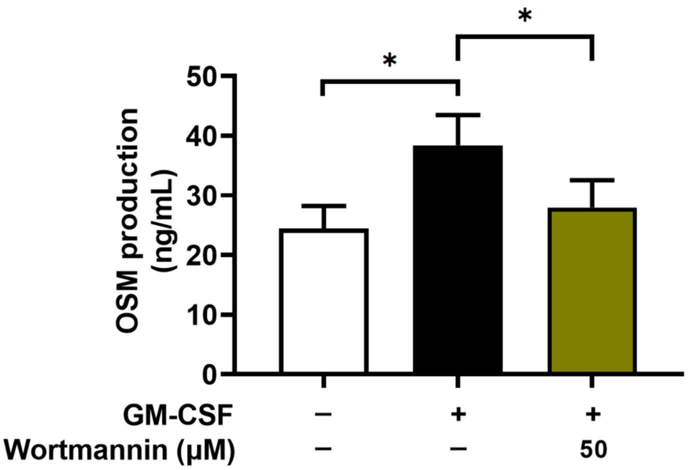

Figure S5. Inhibitory effects on OSM production by Wortmannin (PI3K inhibitor) in dHL-60 cells. The cells (2.5×10^5 cells in 500 μL of medium) were exposed to recombinant human GM-CSF (5 ng/mL) for 4 h after pretreatment with Wortmannin (50 μM) for 1 h. OSM production was measured by ELISA. Data are the mean ± SD of three independent experiments. *$p < 0.05$ signifies significant differences.

Materials and Methods

Cell Viability

dHL-60 cells (5×10^4 cells in 500 μL of medium) were seeded in a 24-well plate and pretreated with NAR or PBS for 1 h, and then stimulated with GM-CSF (5 ng/mL) for 4 h. The cells were incubated with 3-(4,5-dimethylthiazol-2-yl)-2,5-diphenyltetrazolium bromide (MTT, Sigma-Aldrich Co.) solution at 37 °C for 4 h. Next, we added 1 mL of dimethyl sulfoxide (DMSO) to dissolve the MTT formazan, and transferred 100 μL of supernatant into a new 96-well microplate. A microplate reader (540 nm, Versa Max, Molecular Devices, Sunnyvale, CA, USA) was used to measure the absorbance of formazan dissolved in DMSO.

OSM Measurement

dHL-60 cells (2.5×10^5 cells in 500 μL of medium) were seeded in a 24-well plate and pretreated with NAR or PBS for 1 h, and then stimulated with GM-CSF (5 ng/mL) for 4 h. OSM levels were assessed by means of an enzyme-linked immunosorbent assay. The capture antibody (R&D system Inc., Minneapolis, MN, USA) was pre-coated in a 96-well plate. Phosphate-buffered saline (PBS) containing 10% FBS was added to block the plate for 2 h. After washing the plate by means of PBS containing Tween 20 (PBST), cell supernatants were added into the plate for 2 h. After washing the plate with PBST, the plate was treated with biotinylated detection antibody (R&D system Inc.) for 2 h and then incubated with avidin-conjugated to horseradish peroxidase (Sigma-Aldrich Co.) for 30 min. Absorbance by TMB substrate (BD Pharmingen, San Jose, CA, USA) was measured by a microplate reader (405 nm, Versa Max).

Real-Time Quantitative PCR

dHL-60 cells (2 × 10^6 cells in 2 mL of medium) were seeded in a 6-well plate and pretreated with NAR or PBS for 1 h, and then stimulated with GM-CSF (5 ng/mL) for 30 min. The harvested cells were used to isolate total RNA by means of an RNA extraction reagent (iNtRON, Seongnam, Republic of Korea). The first-strand cDNA from total RNA was synthesized with cDNA synthesis reagents (Bioneer, Daejeon, Korea). The following designed primers were used for the real time PCR (Applied Biosystems, Foster City, CA, USA) by using Power SYBR® Green Master Mix (Applied Biosystems): OSM: 5′- GCTCACACAGAGGACGCTG-3′, 5′-GGAGCACGCGGTACTCTTTC-3′; GAPDH: 5′-TCGACAGTCAGCCGCATCTTCTTT-3′, 5′-ACCAAATCCGTTGACTCCGACCTT-3′. The relative expression of mRNA for OSM was normalized by GAPDH and measured by using $2^{-\Delta\Delta Ct}$ method.

Western Blot Analysis

dHL-60 cells (1 × 10^7 cells in 2 mL of medium) were seeded in a 6-well plate and pretreated with NAR or PBS for 1 h, and then stimulated with GM-CSF (5 ng/mL) for 15 min (PI3K) or 30 min (Akt) or 30 min (NF-κB). An ice-cold cell lysis buffer (Sigma-Aldrich Co.) was used to lyse the harvested cells. Cell extracts were prepared with sampling buffer (Laemmli's 2×, ELPISBIOTECH. INC., Daejeon, Korea) and heated at 95 °C for 5 min. Proteins were subjected to electrophoresis using 10% - 15% gel containing sodium dodecyl sulfate and transferred to nitrocellulose membranes (Amersham™, Chicago IL, USA). PBST containing 5% bovine serum albumin (Sigma-Aldrich Co.) was used to block the membranes afterwards relevant primary antibodies (phosphorylated (p)-PI3K, Cell Signaling Technology, Danvers, MA, USA; PI3K, p-Akt, Akt, p-p65, p65, and GAPDH, Santa Cruz Biotechnology, Santa Cruz, CA, USA) were used. Peroxidase-conjugated secondary antibodies (Santa Cruz Biotechnology) were added for incubation of the membranes for 1 h at room temperature after washing with PBST. Specific bands were detected by an enhanced chemiluminescence solution (DoGenBio Co., Seoul, Korea). Band intensities were calculated with the ImageJ program (National health institute, Bethesda, MD, USA).

Immunofluorescence Analysis

dHL-60 cells (2 × 10^6 cells in 2 mL of medium) were seeded in a 6-well plate and pretreated with NAR or PBS for 1 h, and then stimulated with GM-CSF (5 ng/mL) for 30 min. dHL-60 cells were fixed with 4% paraformaldehyde, permeabilized in 0.2% Triton X-100, and incubated with a blocking buffer to reduce nonspecific binding. The cells were incubated with the primary antibody (anti-p-p65), followed by incubation with Alexa Fluor® conjugated secondary antibody (Alexa Fluor® 647) at room temperature. For nuclear staining, 4′ ,6-diamidino-2-phenylindole (DAPI) was used. Samples were visualized under a confocal laser-scanning microscope (Carl Zeiss, Oberkochen, Germany).
